# Antifungal potential of multi-drug-resistant *Pseudomonas aeruginosa*: harnessing pyocyanin for candida growth inhibition

**DOI:** 10.3389/fcimb.2024.1375872

**Published:** 2024-05-22

**Authors:** Mohammad Oves, Mohd Shahnawaz Khan, Majed Al-Shaeri, Mohammad Saghir Khan

**Affiliations:** ^1^ Center of Excellence in Environmental Studies, King Abdulaziz University, Jeddah, Saudi Arabia; ^2^ Protein Research Chair, Department of Biochemistry, College of Science, King Saud University, Riyadh, Saudi Arabia; ^3^ Department of Biological Science, Faculty of Science, King Abdulaziz University, Jeddah, Saudi Arabia; ^4^ Department of Agricultural Microbiology, Faculty of Agricultural Science, Aligarh Muslim University, Aligarh, India

**Keywords:** drug resistance, *Pseudomonas aeruginosa*, pyocyanin, plasmid, antifungal, anti-candida, exopolysaccharide

## Abstract

**Introduction:**

*Pseudomonas aeruginosa* is notorious for its multidrug resistance and its involvement in hospital-acquired infections. In this study, 20 bacterial strains isolated from soil samples near the Hindan River in Ghaziabad, India, were investigated for their biochemical and morphological characteristics, with a focus on identifying strains with exceptional drug resistance and pyocyanin production.

**Methods:**

The isolated bacterial strains were subjected to biochemical and morphological analyses to characterize their properties, with a particular emphasis on exopolysaccharide production. Strain GZB16/CEES1, exhibiting remarkable drug resistance and pyocyanin production. Biochemical and molecular analyses, including sequencing of its 16S rRNA gene (accession number LN735036.1), plasmid-curing assays, and estimation of plasmid size, were conducted to elucidate its drug resistance mechanisms and further pyocynin based target the *Candida albicans* Strain GZB16/CEES1 demonstrated 100% resistance to various antibiotics used in the investigation, with plasmid-curing assays, suggesting plasmid-based resistance gene transmission. The plasmid in GZB16/CEES1 was estimated to be approximately 24 kb in size. The study focused on *P. aeruginosa*’s pyocyanin production, revealing its association with anticandidal activity. The minimum inhibitory concentration (MIC) of the bacterial extract against *Candida albicans* was 50 μg/ml, with a slightly lower pyocyanin-based MIC of 38.5 μg/ml. Scanning electron microscopy illustrated direct interactions between *P. aeruginosa* strains and *Candida albicans* cells, leading to the destruction of the latter.

**Discussion:**

These findings underscore the potential of *P. aeruginosa* in understanding microbial interactions and developing strategies to combat fungal infections. The study highlights the importance of investigating bacterial-fungal interactions and the role of pyocyanin in antimicrobial activity. Further research in this area could lead to the development of novel therapeutic approaches for combating multidrug-resistant infections.

## 1 Introduction

One of the most significant microorganisms in intensive care units is *Pseudomonas aeruginosa*, known for causing nosocomial infections and sepsis in wounds upon acquiring virulence plasmids (Hu et al., 2021). In the past two decades, *Pseudomonas* infection has become a leading cause of hospital infections, responsible for 20% of all cases (Reynolds and Kollef, 2021). *P. aeruginosa* is an opportunistic pathogen, and its contamination is highly vulnerable in developing countries because vast numbers of people die due to lack of medicine and incurable infections (Akram et al., 2022). *P. aeruginosa* is the most prevalent nosocomial pathogen. It quickly targets hospitalized patients, and an extended stay period leads to the colonization of organisms in organs and increases the risk of mounting a deadly infection (Wood et al., 2023). The other serious bacterial infections are pneumonia, septicemia, malignant external otitis, endophthalmitis, and endocarditis, which are also related to the primary *Pseudomonas* infection (Denissen et al., 2022; Zhang et al., 2024). Sometimes, ordinary people acquire infections through hospital air, walls, toilets, and medical equipment like needles or catheters (Abreu et al., 2013). Patients who require mechanical ventilation are at a heightened risk of developing nosocomial pneumonia, a complication similar to that of burn victims (Wu et al., 2019). This increased susceptibility also increases the likelihood of contracting *Pseudomonas* infections, which may extend to urinary tract infections and blood-borne diseases (John et al., 2016). In immunocompromised patients, however, strains of *P. aeruginosa* can cause severe infections throughout the body. *P. aeruginosa* infections are most prevalent in immunocompromised patients with conditions such as cystic fibrosis, organ transplants, leukemia, or intravenous drug use (Jackson and Waters, 2021; Paprocka et al., 2022).

Early eradication of *P. aeruginosa* infections is crucial for immunocompromised patients, as established infections can prove difficult to eliminate and lead to higher mortality and morbidity rates (Reynolds and Kollef, 2021). Cystic fibrosis patients, in particular, are prone to lung infections caused by *P. aeruginosa*, which is a leading cause of illness and death in these patients (Malhotra et al., 2019).

P. aeruginosa is also associated with keratitis, a common affliction among contact lens wearers (Teweldemedhin et al., 2017). In addition, exposure to *P. aeruginosa* contaminated water can result in ear infections and folliculitis (Araos and D’Agata, 2019). *Pseudomonas aeruginosa* plays a role in sepsis and regulates inflammation and autophagy downregulation (Jabir, M. et al., 2015).

The indicators of a *P. aeruginosa* infection are contingent upon the specific type and location. Localized wound infections usually manifest with blue-green pus close to the affected area. In contrast, infections in the respiratory system may display symptoms such as pneumonia, including cough and fever. Bloodstream infections can result in severe symptoms such as high fevers, chills, and septicemia (Huerta and Rice, 2019). The excessive utilization and misuse of antibiotics have resulted in widespread resistance to these medications, making it increasingly challenging to treat bacterial infections with conventional treatments (Uddin et al., 2021). Antibiotics usually go after two main ways that Gram-negative bacteria cross their outer membrane: a lipid-mediated channel for antibiotics that do not like water and diffusion by porin for antibiotics that do. The composition of lipids and proteins in the outer membrane significantly influences the development of drug resistance (Zhang et al., 2021).

*Pseudomonas aeruginosa* has a double-cell membrane as a Gram-negative that contains prions, which act as an efflux pump, contributing to drug resistance. This mechanism effectively pumps out most antibiotics before they reach their target site within the cell (Delcour, 2009). This grouping of bacteria possesses intrinsic resistance to most beta-lactam-based antibiotics, attributable to their exceptionally low cell wall permeability.

*Pseudomonas aeruginosa*, a motile organism belonging to the family Pseudomonadaceae and possessing a single polar flagellum, is a ubiquitous bacterium in various environments. It can develop multidrug resistance through genetic mutations, phase variation, and horizontal gene transfer of resistant genes (Silva et al., 2020). One way *P. aeruginosa* gets around antibiotics is by making an inducible cephalosporinase enzyme. This enzyme increases active efflux and lowers its affinity for the target site in DNA gyrase, which makes the cell wall more permeable (Langendonk et al., 2021). Biofilm formation leads to phenotypic resistance and the growth of small colony variants, which are both important for drug resistance during antibiotic treatment (Yamani et al., 2021). Pyocyanin is a secondary metabolite that the opportunistic pathogen *Pseudomonas aeruginosa* produces and is well-known for its biological activities, including its antibacterial properties (Houshaymi et al., 2019). Pyocyanin’s capacity to trigger oxidative stress and interfere with vital cellular functions has attracted attention for its potential therapeutic uses (Hall et al., 2016). A recent study has concentrated on investigating the effectiveness of pyocyanin against different microbial diseases, such as fungi like Candida species (Abd El-Baky et al., 2020). Candida, especially *Candida albicans*, is a prevalent opportunistic fungal pathogen that can lead to various diseases, ranging from superficial mucocutaneous infections to invasive bloodstream infections with significant fatality rates. The rise of drug-resistant Candida species highlights the immediate requirement for new antifungal medicines and treatment approaches (Peyclit et al., 2021). Exploring the ability of pyocyanin to target Candida is a viable direction for developing new antifungal treatments in this scenario. Studying the relationship between pyocyanin and *Candida* and uncovering the mechanisms of action could lead to new strategies to fight fungal infections and address the increasing problem of antibiotic resistance.

This study aimed to isolate antibiotic-resistant bacteria from a polluted soil sample from the Hindon River near New Delhi, India. Standard techniques tested the drug resistance of the plasmid and the transfer of antibiotic-resistance genes to recipient bacteria. Furthermore exploring exopolysaccharide synthesis by the stain and pyocyanin synthesis was also optimized and employed as a potent anti-candida agent.

## 2 Materials and methods

### 2.1 Soil sample collection

Soil samples for this study were collected from lands irrigated by the Hindan River, flowing through Ghaziabad district’s industrial area near the National Capital Region (NCR), India. This region is characterized by diverse industrial activities, including chemical, food, metal processing, and paint manufacturing, where sewage wastewater is often discharged into the river. The geographic coordinates for the water sample collection site are 28.67°N latitude and 77.42°E longitude.

### 2.2 Isolation of bacteria

A composite mixture of soil samples was refined with a sieve weight of up to 1 g and serially diluted with normal saline solution. These serially diluted samples were poured 0.1 ml on Kings B agar media plates and left to grow at 35°C for 48 hours in an incubation chamber. Kings B media comprises protease peptone (20 g/l), K_2_HPO_4_ (2.4 g/l), MgSO_4_.7H_2_O (6 g/l), SDS (0.6g), and glycerol (10 ml/l). Following 48 hours of incubation, many colonies were observable on the surface of the media plate; however, only those displaying blue and green pigmentation were selected for culture purification. These pure cultures were subsequently stored in nutrient broth supplemented with 15% glycerol and employed for further analysis in this study.

#### 2.2.1 Colony morphology and Gram staining

The characteristics and colony morphology of the isolated bacterial culture were examined, and a Gram stain was conducted. The results indicated that the purple-colored colonies were gram-positive, while those in pink were classified as gram-negative (More detailed biochemical test present in a **Supplementary File**).

#### 2.2.2 Molecular identification of bacterial strain

Among the 20 bacterial isolates, the strain exhibiting the highest level of antibiotic tolerance was selected for species identification. Analyzing the 16S rRNA gene’s most stable, unchanging region helped achieve this. The selected strain was obtained from a muddy soil sample collected at a heavily polluted site along the edge of the Hindan River in Ghaziabad, India. The 16S rRNA gene sequence from Macrogen’s online sequencing system, South Korea Inc., which offers paid sequencing services after the submission of the whole microorganism. Universal primers (518F and 800R) were used to amplify the target gene and standard PCR protocols were followed for amplification of target sequence (Park et al., 2021). The sequence was reported to the GenBank database through an online tool, and a unique access code was also obtained for further analysis. The originality of the sequence was confirmed using BLASTn, an online program that compares the isolate with the most closely related sequences available in the database of NCBI (http://www.ncbi.nlm.nih.gov/BLAST). This program uses maximum likelihood sequences and previous genomic and taxonomic information to identify and compare the sequence with databank accurately. Additionally, the most closely related sequences were aligned with the original sequence using the CluastalW online program and MEGA7.2 software (Thompson et al., 1994). A standard neighbor-joining (Nj) method was used to develop the phylogenetic tree. The MEGA7.2 software online program assessed bootstrapped neighbor-joining relationships (Tamura et al., 2007).

### 2.3 Antibiotic profiling of bacterial isolates

The researchers utilized a standard procedure for disc diffusion to assess the antibiotic sensitivity of isolated bacterial strains. Antibiotic discs of known efficacy (**Supplementary Table 1**) were obtained from the Hi-Media Laboratory in Mumbai, India. Before the study, freshly prepared medium plates were incubated overnight at 35°C without antibiotic discs or bacterial inoculum to ensure sterility. Each isolate was then inoculated in freshly prepared nutrient broth using a single colony from an agar slant, which was then incubated at 35°C overnight to obtain a new culture. From the fresh culture, 100 µl of test culture was taken and transferred onto the surface of solid agar media plates and spread with a sterile L-shaped glass rod. After 5 minutes, antibiotic discs were placed on the surface of agar plates using sterile forceps, and the plates were placed for overnight incubation at 35°C. After overnight incubation, plates were assessed and measured for a zone of inhibition. According to the bacteria strain potential, it was recorded as resistant (R) and susceptible (S) according to the standard method (Margalejo et al., 1984). The pattern of resistance or sensitivity of the bacterial strain on the plates was recorded and compared with the zone of inhibition illustration delivered by the disc manufacturer (HiMedia).

### 2.4 Plasmid isolation of antibiotic-resistant bacterial isolates

It was found that the antibiotic resistance trait was present on the plasmid DNA of either strain GZB16 of *P. aeruginosa* (Bimboim and Doly, 1979). The DNA was extracted using an alkaline lysis protocol. Following extraction, the DNA was examined using 0.8% agarose gel electrophoresis. We ran the gel horizontally at 60 mA for two hours with a 500-mL standard buffer solution that had a pH of 8.3 and was made up of 1 mM Tris-Borate-EDTA (TBE — 1). After the gel electrophoresis, a 0.5 µg/ml ethidium bromide solution was used to color the gel for 5 minutes. The gel pattern could then be seen with a UV transilluminator. Images were captured using a gel-documentation system (Model G: BOX, Syngene). A standard plasmid marker from *E. coli* DNA was used to examine the molecular weight of the plasmid. We used DNA cut with EcoRI and Hind III to find the molecular weight and as a guide for the agarose gel electrophoresis. It was run at the same time as the plasmid DNA from the *Pseudomonas strain*.

#### 2.4.1 The plasmid curing assay

The present technique utilizes a physical approach to eradicate plasmids from highly resistant bacterial isolates. Initially, fresh bacterial cultures were cultivated as previously described, and subsequently, the bacterial cells were subjected to a treatment method at 45°C, as reported by Fortina and Silva (1996). In summary, individual bacterial isolates were inoculated into two nutrient broth-containing flasks. One flask was incubated at the optimal temperature of 37°C. In contrast, the other was incubated overnight at the optimal temperature and subsequently elevated to 45°C, eliminating the plasmid from the bacterial cells. The bacterial strain was then incubated with 5% acridine orange to enhance accuracy. The efficacy of the plasmid curing process was verified through antibiotic susceptibility testing. A standard bacterial stain was used to confirm the plasmid curing from the bacterial cells.

#### 2.4.2 Plasmid transformation

The transformation of plasmids is achieved through conjugation, wherein *P. aeruginosa* acts as the donor, and a conventional *E. coli* culture acts as the recipient during incubation. The *E. coli* strain used as the recipient is of the standard variety and is rifampicin resistant (ATCC 25922 Rif). Agar media with Gm (20 µg/mL) +Rif (80 µg/mL) and CTX (25 µg/mL) + Rif (75 µg/mL) was utilized to select the transconjugant strain. A special kind of *E. coli* cell called recA was put in a room with 260 nm UV light and ethidium bromide or acridine orange at levels that did not stop the cell’s growth completely (Clark et al., 1994). 200 µL of ice-cold 50 mM CaCl_2_ was added to 500 µL of log-phase bacterial suspension. The culture was incubated in the cold for 30 min using the modified method (Malik and Aleem, 2011). This made the cells ready to be transformed. The bacterial culture suspension was centrifuged at 10,000 rpm for 10 minutes, the pellet was selected, and the supernatant was discarded. For the second time, 250 µL of ice-cold 50 mM CaCl_2_ solution was added to the cell pellet. Then, 500 µL of the extracted plasmid solution was added. After centrifuging the tubes, they were kept warm for 10 minutes at 25°C. Then, 100 µL portions were put on a selective media plate and spread out evenly (on media plates with 80 µg/ml CTX and 20 µg/ml GM, respectively) on a non-selective medium. The cultured plates were then incubated at 35°C overnight to observe the appearance of colonies and further tested for the presence of the transformed plasmid.

### 2.5 Exopolysaccharide production

In this experiment, the bacterial strains were cultivated in 100-ml flasks containing a basic medium enriched with 5% sucrose. The inoculated substance’s flasks were placed in an incubator and kept at 30 ± 2°C for five days. The incubator was equipped with a rotary shaker set at a speed of 120 rpm. The culture was centrifugated at a force of 5724g for 30 minutes. Three cooled acetone (CH_3_COCH_3_) parts were mixed with one part of the supernatant to make the extracellular polymeric substance (EPS). The EPS formed due to precipitation was washed multiple times, alternating between distilled water and acetone. It was then transferred to filter paper No. 42, dried in an oven, and weighed (Bhat et al., 2020).

### 2.6 Bacterial biomass and pyocyanin quantification

The *P. aeruginosa* microbial biomass and pyocyanin content were measured periodically during the fermentation process. The bacterial biomass was obtained by subjecting the fermented broth to centrifugation at a force of 5000 times the acceleration due to gravity for 10 minutes. The resulting biomass was washed twice using sterile distilled water and oven-dried at 80°C overnight.

To determine if prodigiosin was present, 2.5 mL of the liquid part of the cells was taken out and mixed with 1.5 mL of chloroform and 0.5 mL of a solution containing 0.2-N hydrochloric acid. According to Essar et al., researchers could determine how much pyocyanin was in the cell-free supernatant by measuring its absorbance (A) at 520 nm in its acidic form (Essar et al., 1990). The quantity of pyocyanin was determined using the subsequent formula: The concentration of pyocyanin (mg/L) is calculated by multiplying the absorbance at 520 nm (A_520_) wavelength by the conversion factor 17.072.


$$Pyocyanin\;(mg/L)=A_{570}\;x\;17.072$$

### 2.7 Anti-candida activity

#### 2.7.1 Zone inhibition assay

The zone inhibition assay was performed according to the standard method of the Clinical & Laboratory Standards Institute (CLSI) guidelines because this test is rapid, simple, and affordable to test the antimicrobial susceptibility. A stock solution of pyocyanin was developed from the bacterial extract after the 96 h incubation. Initially, develop the Sabouraud’s Dextrose agar (SDA) plates using the standard sterile method. A fresh culture of *Candida albicans* 0.1 *ml* was poured on the SDA plates and spread by the sterile glass rod spreader. We waited for a dry spreading culture on the plate and made a well on the plate. Each well was sealed in the bottom by molten agar and loaded with the antibacterial testing agent pyocyanin in wells. After this, these plates were placed in the incubator for 48 hours at 30°C. The pyocyanin antimicrobial efficiency was determined by measuring the zone inhibition caused by the testing agent.

#### 2.7.2 Biofilm experiment

##### 2.7.2.1 Fresh bacterial culture preparation

From the culture tube, 0.1 ml culture inoculates *Pseudomonas aeruginosa* isolates into fresh LB 100 ml broth and incubates overnight at 37°C and agitation for good growth.

##### 2.7.2.2 Fresh *Candida albicans* culture preparation

From the cultured Patri plate, a full loop of the culture of Candida strains was inoculated into Sabouraud dextrose broth and incubated overnight at 37°C temperature for growth.

According to Christensen et al.’s description of the methodology, this study uses a qualitative approach to biofilm identification (Christenson and Sims, 2012). Specifically, 5 ml of Trypticase soy broth was inoculated with a small amount of microorganisms using a loop and then incubated for 24 hours at 37°C. The tubes were then poured off and rinsed with phosphate buffer saline (pH 7.2) before drying in the air. Subsequently, the tubes were dyed with crystal violet (0.1%) for 15 minutes and rinsed with deionized water. Finally, the tubes were allowed to air dry while positioned upside down. The formation of biofilms was assessed using control strains. Organisms were considered to produce biofilms if a visible layer was present on the walls and base of the tube. If a ring was observed at the interface of the liquid medium, the organism was deemed incapable of biofilm production. The experiment was repeated three times.

The wells of a 96-well flat-bottom polystyrene tissue culture plate from Thermo Fisher Scientific in Shanghai, China, were filled with 0.2 ml portions of diluted culture, which were then incubated at 37°C for 24 hours. After incubation, the contents were removed from the plates by gently tapping. The plates were rinsed twice with 0.2 ml of phosphate-buffered saline (pH 7.2) and then incubated at 37°C for one hour. 0.2 ml of 0.1% crystal violet was applied to the plates and left for 15 minutes.

The excess amount of dye with culture was released by washing the plates twice with deionized water, and then they were allowed to dry. Two hundred microliters of a 33% glacial acetic acid solution were added to the wells. The optical density (OD) of the isolates was measured using a micro ELISA auto reader (BIORAD 680) at a wavelength of 570 nm (OD 570 nm). The experiment was repeated three times.

#### 2.7.3 Pyocyanin effect on candida survivability and biofilm

A fresh culture of Candia albicans was treated with different doses of pyocyanin extract from the bacterial culture incubated at 0 to 120 h. After inoculation, each flask was placed in a rotating incubator, and the growth of candida albicans appeared as a colony on the SDA plate and calculated CFU and represented the survival percentage in the presence of pyocyanin.

Initially, the biofilm of Candida was effectively cultivated in a microtiter plate by adding Sabouraud’s dextrose broth. The biofilm levels were further evaluated upon exposure to varying concentrations of pyocyanin. To measure the biofilm quantitatively, the biofilm was thoroughly rinsed three times with phosphate-buffered saline without disturbing the biofilm in each well. Then, a 0.1% w/v ethanol solution of crystal violet was added drop by drop to each well and incubated for 20 minutes. Subsequently, the staining solution was eliminated, and the wells were delicately rinsed with distilled water. Afterward, two milliliters of ethanol were introduced into each well to dissolve the crystal violet that had adhered. The solution was carefully stirred to ensure full dissolution. The spectrophotometer was used to measure the absorbance of the resultant solution within the wavelength range of 570–590 nm. Relative biofilm formation is calculated by the correlated absorbance of the treated sample divided by the correlated absorbance of the control sample of the biofilm.

#### 2.7.4 Targeting candida cell by *Pseudomonas aeruginosa*

The overnight-grown fresh culture of *Pseudomonas aeruginosa* and fresh culture of *Candida albicans* were mixed in a 1:2 ratio and incubated in the incubator for two hours at 30°C. After the incubator, a 2 ml sample was transferred into the Eppendorf, washed with phosphate buffer, fixed with glutaraldehyde (2%) solution, and placed in the refrigerator at 4°C temperature for overnight. After overnight fixing, the microbial sample was washed with normal saline solution and 10 to 80% ethanol solution. After drying, the sample was mounted on the stab of scanning electron microscopy, and the sample was viewed at 8000X magnification at 20 kV.

## 3 Results

### 3.1 Isolation of drug resistance *Pseudomonas* stain

**Figure 1:**
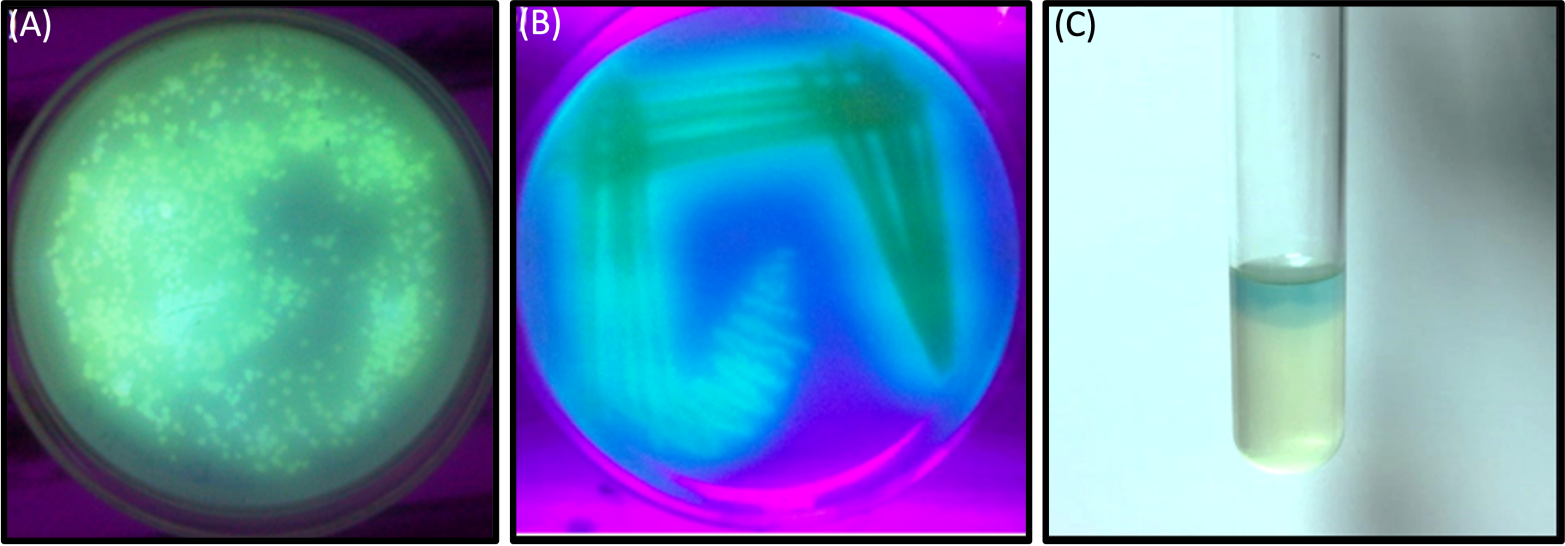
In a culture incubator at 35°C, the *Pseudomonas aeruginosa* bacterial strain displayed a greenish pyocyanin pigmentation when exposed to ultraviolet light after a 3-days. **(A)** A plate showing the bacterial colony on Kings B agar plates, with the characteristic greenish pigmentation visible under ultraviolet light. **(B)** A purified bacterial culture demonstrating fluorescent pigmentation on a medium plate, with the pigmentation appearing greenish under ultraviolet light. **(C)** A liquid medium containing the bacterial strain GZB16/CEES1 exhibits the distinctive greenish pigmentation of pyocyanin.

In this investigation, selected twenty soil samples from diverse locations along the Hindon River in the National Capital Region and the outskirts of Ghaziabad, near New Delhi, India. Subsequently, we isolated the most antibiotic-resistant bacterial strain from each sample and designated it GZB1 to GZB20. All of the recovered bacterial isolates were confirmed to belong to the *P. aeruginosa* species on the King’s B agar plate (as illustrated in **Figure 1A**). This bacterial isolate was cultured in King’s B media supplemented with glycerol, which exhibited a fluorescent green color under UV light, as shown in the figure (**Figure 1B**). Our findings indicate that among the 20 samples analyzed, GZB 16/CEES1 displayed the highest resistance level (100%) to the antibiotics tested. In contrast, GZB 1 and GZB 2 exhibited the lowest resistance (35%) (**Supplementary Figure 1**; **Supplementary Table 3**). The isolated bacterial strains were gram-negative, small rod-shaped, arranged in single short chains, motile, non-spore-forming, and pigment-producing. Employing a combination of microscopical, morphological, biochemical, and molecular sequencing, we successfully isolated and identified all strains of *P. aeruginosa*. The most resistant isolate, GZB16/CEES1, was characterized by using 16 S rRNA gene sequencing technique. The strains were grown on solid and liquid media and observed for pigment production, with all isolates producing green pigment when grown on King’s media supplemented with glycerol at 37°C. This pigment is known as pyocyanin (**Figure 1C**). The biochemical tests showed that most strains were positive for citrate, oxidase, catalase, lipase, and urease production. On the other hand, they were negative for tests like indole, methyl red, and the Voges-Proskauer (more details in the **Supplementary File**).

### 3.2 Identification and phylogenetic tree construction

**Figure 2:**
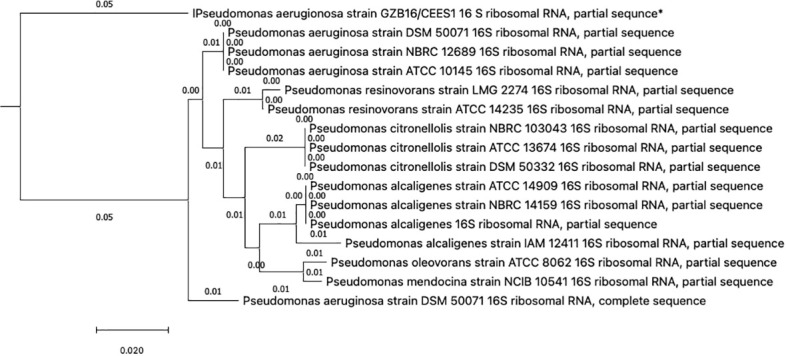
The Neighbor-Joining method constructed the evolutionary relationships of GZB16/CEES1 strains of *Pseudomonas aeruginosa*. The phylogenetic tree was created according to the scale and branch lengths in the same units. The analysis involved nucleotide sequences, and gaps and missing data were eliminated in all positions. Evolutionary analyses were conducted in MEGA7.

This study conducted a preliminary analysis of isolated bacterial strains through biochemical and morphological characterization. Subsequently, they focused on the most promising and multi-drug-resistant bacterial strain, GZB16/CEES1, and investigated it using molecular methods. The *Pseudomonas* strain was first found using biochemical tests that showed it responded well to catalases, oxidases, lipases, urease, citrate utilization, and gelatin hydrolysis. However, it tested negative for methyl red, Voges-Proskauer, lysine decarboxylase, and phenylalanine deaminase. Fluorescent green pigmentation on solid agar media plates, liquid broths, and additional biochemical tests suggested a possible correlation with Pseudomonas aeruginosa. The researchers used the Macrogen Sequencing Service to do 16S rDNA sequencing to confirm that the GZB16 strain was who they said it was. The sequence they got is shown in (**Supplementary Figure 1**) BLASTn sequence analysis revealed 89.36% identity similarity with the *Pseudomonas aeruginosa* strain SDM 50071 (NR 117678.1). The researchers made a phylogenetic tree with maximum-likelihood strain sequences and clustalW alignment to learn more about how this strain is related to other similar strains (**Figure 2**).

### 3.3 Antibiotic profiling of resistance isolate

**Figure 3:**
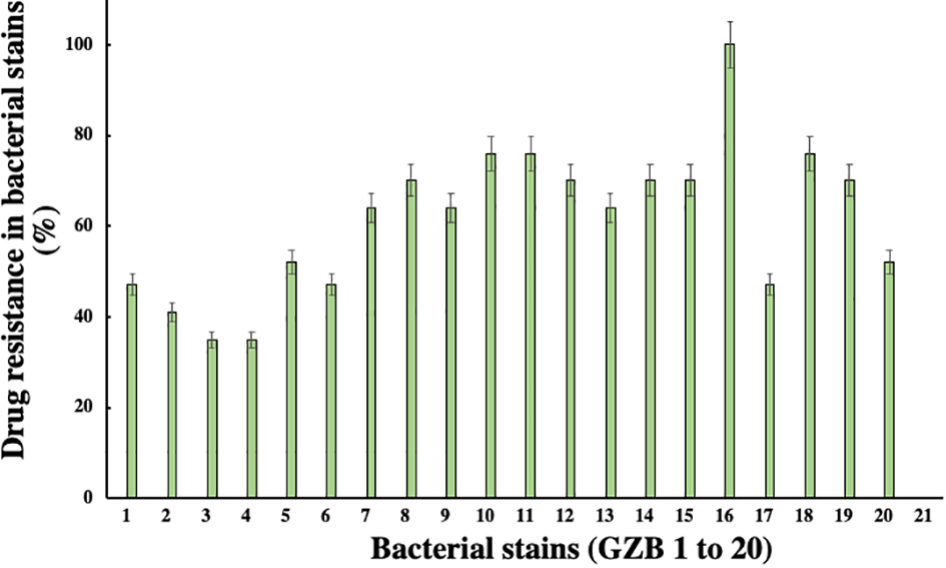
Susceptibility patterns of bacterial strains against the applied total antibiotics.

In this current investigation, a significant proportion of drug-resistant bacterial isolates, exceeding 30%, were obtained from the soil samples collected near the edge of the Hindon River in Ghaziabad, NCR region, India. Among these isolates, strain GZB16/CEES1 demonstrated a remarkable degree of resistance against all 17 antibiotics tested, including Am, CF, CX, C, E, G, K, M, NA, P, Pb, R, Nf, Nx, Do, T, and Nv, with a 100% resistance rate. Strains GZB10, 11, and 18 followed closely, with a resistance rate of 76% against the majority of the antibiotics tested (**Figure 3**). In contrast, strains GZB2 and 3 demonstrated a lower resistance rate of 35% against the antibiotics used in this study. Notably, strain GZB16 also exhibited resistance against imipenem and carbapenem antibiotics up to 30 µg/ml. All strains tested in this study demonstrated resistance against the antibiotics Im, Cx, M, P, and Nf at a fixed dose of 30 µg/ml in liquid media. However, the susceptibility of the strains varied significantly against most antibiotics, and the resistance rates did not exceed 50% in fixed-dose responses (**Supplementary Table 3**). The primary mechanism of antibiotic resistance in strain GZB16 of *P. aeruginosa* was the production of β-lactamase.

### 3.4 The plasmid isolation, curing, and transformation

**Figure 4:**
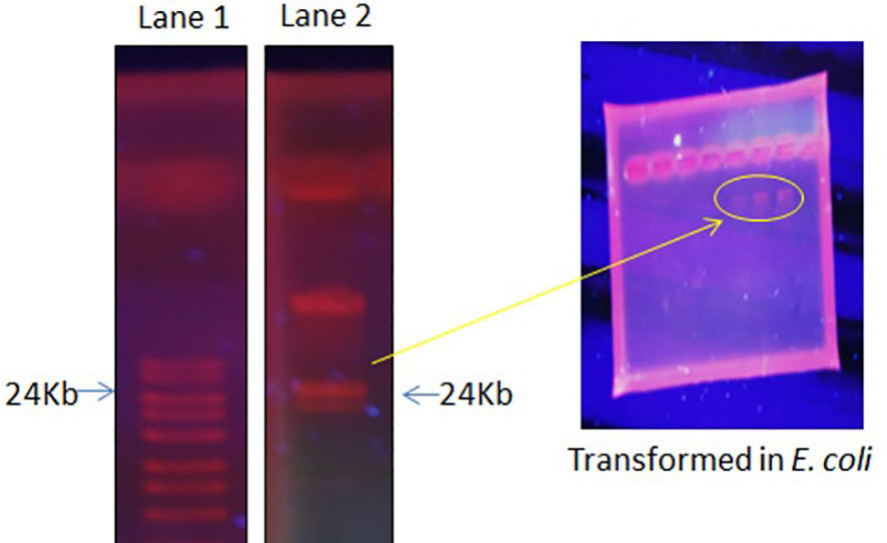
Plasmid DNA isolation and purification by gel electrophoresis analysis, Lane 1 shows the ladder, and Lane 2 reveals the plasmid band. Further, these plasmid bands appeared in the transformed *E. coli* cells when incubated with *P. aeruginosa*.

**Figure 5:**
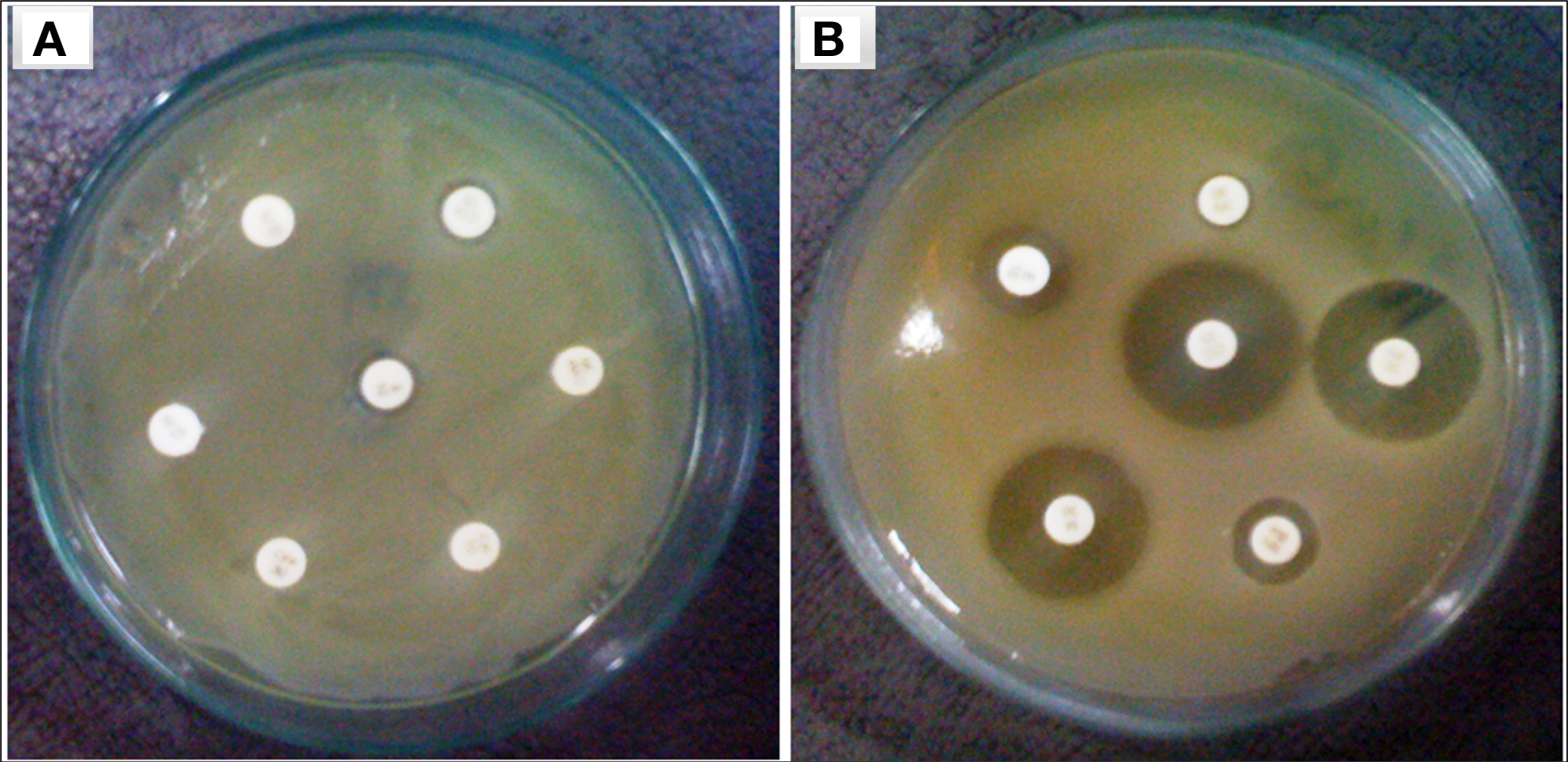
Bacterial strain of *P. aeruginosa* **(A)** plasmid containing bacterial strain showed resistance against multiple antibiotic discs **(B)** without plasmid bacteria stain highly sensitive against similar antibiotics.

The focus of the present investigation was the isolated multidrug-resistant bacterial strain, *P. aeruginosa* GZB 16. The multidrug resistance properties of this strain may be intrinsic or the result of conjugation with other drug-resistant bacterial strains via plasmids. To screen the plasmids responsible for multidrug resistance in the *Pseudomonas aeruginosa* strain, we isolated and analyzed its DNA. As a size marker, we used agarose gel electrophoresis with Eco-RI and Hind-III-digested λ DNA. The isolated plasmid DNA molecular weight was estimated to be approximately 24 kb (**Figure 4**). Conjugation was used to transfer the resistance plasmid gene from *P. aeruginosa* GZB16 to the recipient *E. coli* bacterial strain, which was confirmed through the drug resistance pattern on solid agar plates using a disc diffusion assay. Plasmid curing was then achieved through a temperature variation incubation method, and to enhance accuracy, 5% acridine orange was also used. The curing efficiency was verified through antibiotic susceptibility testing (**Figure 5**). Overall, this study utilized temperature-variation incubation from 35 to 45°C and 5% acridine orange to obtain complete curing of the antibiotic-resistant gene.

### 3.5 Exopolysaccharide production

**Figure 6:**
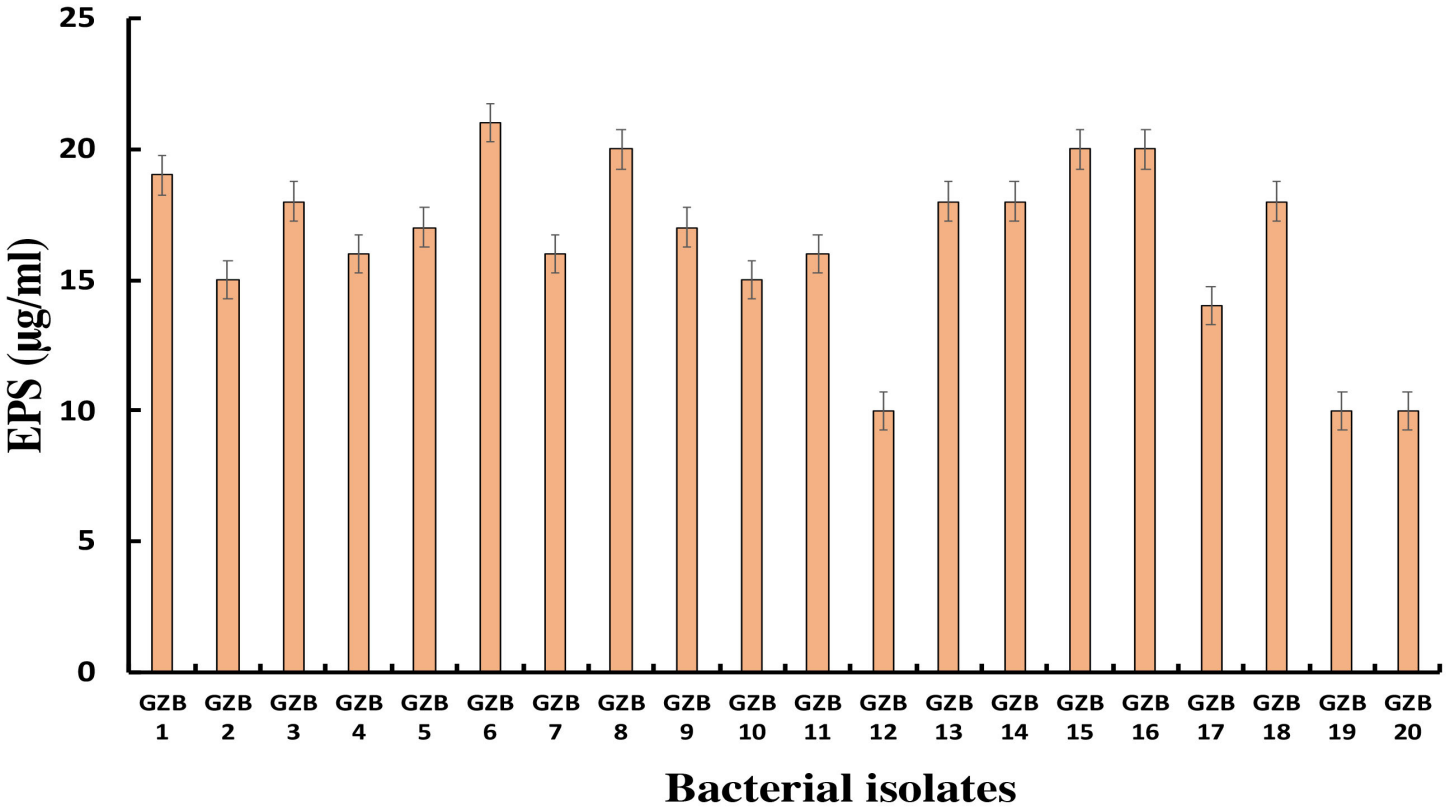
Exopolysaccharide synthesis by *Pseudomonas* isolates at 5% sucrose as an external C- source.

Most bacterial isolates in this study formed exopolysaccharides during their incubation in 5% sucrose, which served as both a carbon source and an intrinsic characteristic. Exopolysaccharide molecules play a crucial role in biofilm formation on any surface by adhering to the surface and protecting cells from negative circumstances. In this investigation, strain GZB 6 was found to be the most prolific producer of exopolysaccharides, with a concentration of 21 µg/ml. Strain GZB 8, followed by strains 15 and 16, each produced 20 µg/ml (**Figure 6**).

### 3.6 Bacterial biomass and pyocyanin quantification

**Figure 7:**
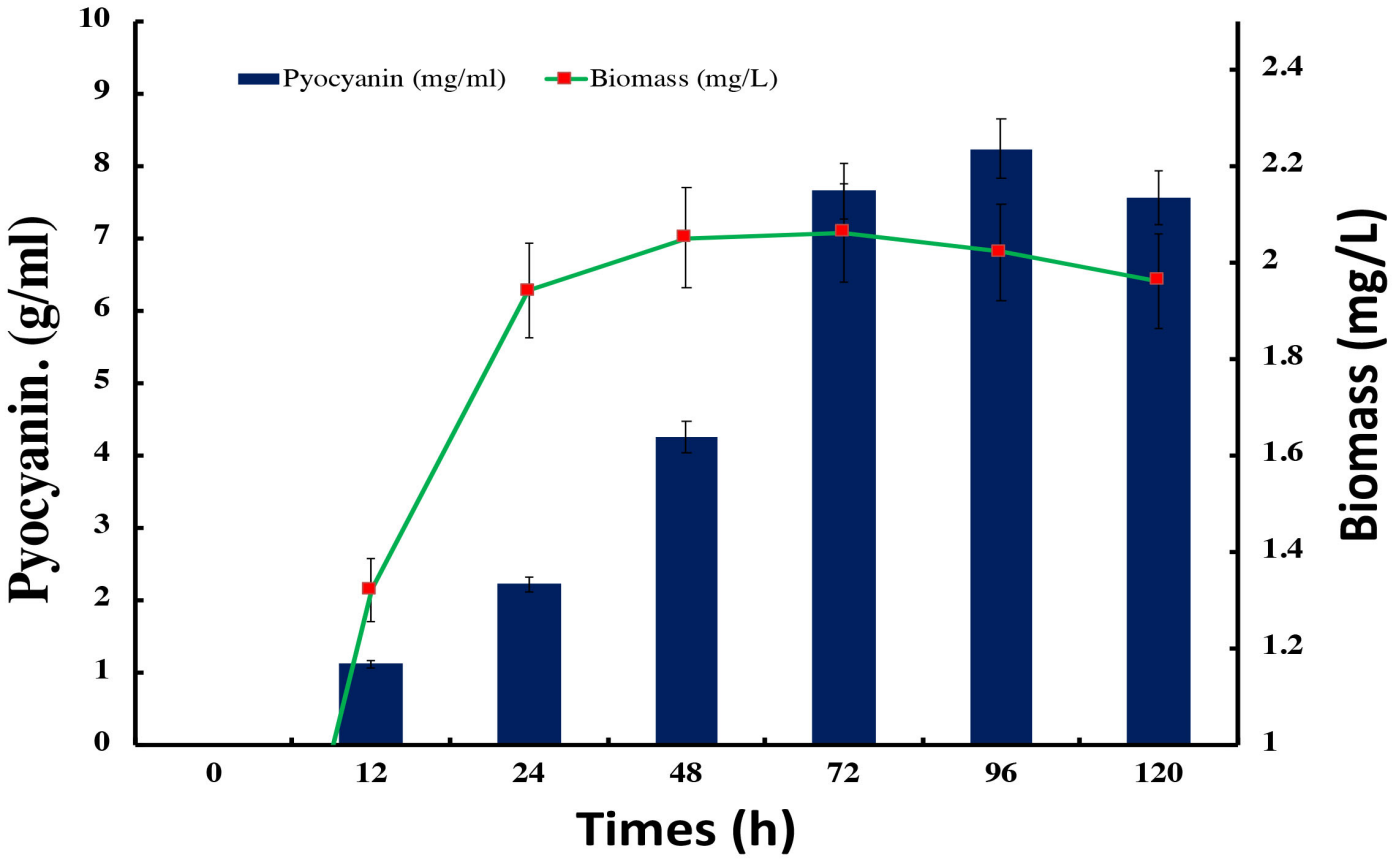
Bacterial pyocyanin synthesis with bacterial biomass at optimum condition.

The relationship between the quantity of bacterial biomass, the progression of bacterial growth in the broth, and the formation of pyocyanin was significant. The bulk of bacterial development in the batch culture medium showed a substantial increase at an appropriate concentration of the current nutrient. However, it suddenly diminished or remained stagnant, indicating that the bacterial strain had reached the stationary phase. During the process of calibrating the synthesis of *Pseudomonas* pyocyanin with bacterial growth, a maximum level of pyocyanin of 7.65 and 8.23 mg/ml was observed after 72 and 96 hours of incubation, respectively, with the maximum biomass levels of 2.15 and 1.91 mg/L (as depicted in **Figure 7**).

### 3.7 Anti-candida activity

#### 3.7.1 Zone inhibition assay

**Figure 8:**
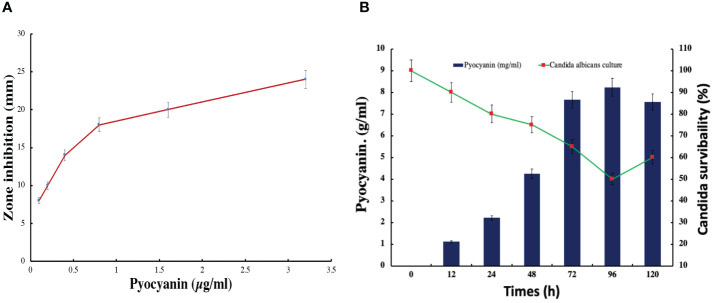
Pyocyanin effect on the candida albicans growth in **(A)** zone inhibition assay **(B)** pyocyanin production and effect on candida survivability.

In this study, the bacterial strain of *Pseudomonas aeruginosa* has significantly synthesized a green fluorescent pigment, pyocyanin, which reveals an excellent antifungal or anti-candida activity. The maximum zone of 24 mm inhibition was observed against *Candida albicans* in the presence of 3.2 µg/ml pyocyanin (**Figure 8**). The zone of inhibition was increasing with concentration in the well of plates. Further, check the *Candida albicans* survivability in the presence of pyocyanin produced by *Pseudomonas* after different incubation periods. The minimum candida serviceability was observed after 96 h incubation because of the high production of pyocyanin. Further survivability increased due to the decreased phase of bacteria and a lesser amount of pyocyanin being produced than in the stationary phase (**Figure 8**).

#### 3.7.2 Effect on candida biofilm and survivability

**Figure 9:**
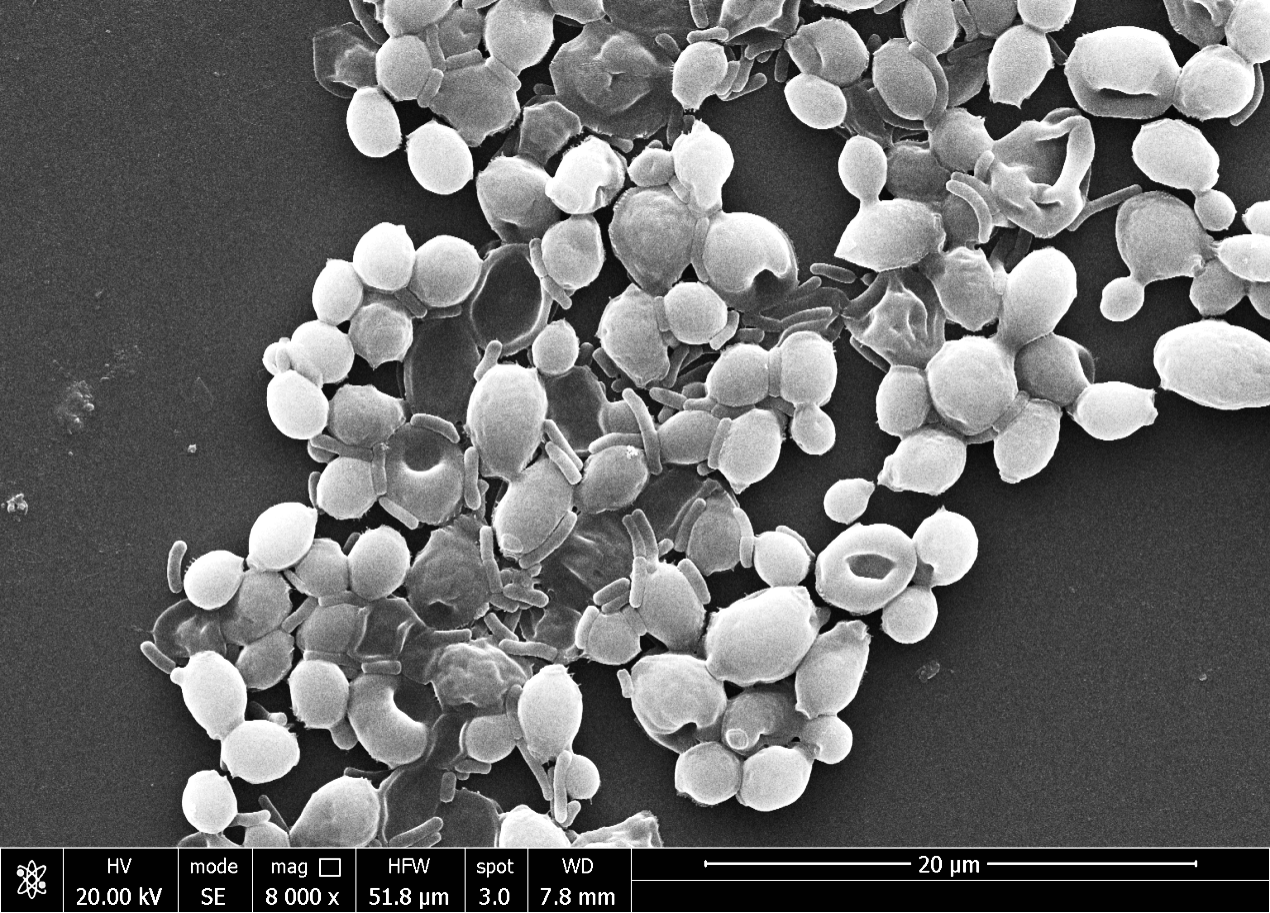
The scanning electron microscope image reveals the *Pseudomonas aeruginosa* bacterial cells directly attached to the *Candida albicans* cells and damaged them.

In this study, *Candida* biofilm formation is significantly inhibited by *Pseudomonas* pyocyanin production when bacterial pyocyanin is amended in the candida-growing wells. The *Candida albicans* survivability is 100% with zero pyocyanin production and solvability directly affected by the pyocyanin production by *Pseudomonas* bacterial culture. The maximum pyocyanin production was observed at 96-hour incubation, and then candida growth decreased up to 50% in the growing media (**Figure 8B**). When a fresh bacterial culture and *Candia albicans* in a 1:2 ratio incubate together, bacterial cells directly attached to the *Candida* cell, directly the candida cells and break the cell growth. This activity was revealed by scanning electron microscopy (**Figure 9**).

## 4 Discussion

The muddy soil samples were collected from the banks of the Hindan River, Ghaziabad, the National capital region near New Delhi, India. The assessment of antibiotic resistance in the 20 bacterial isolates (GZB 1 to GZB 20) obtained from the water samples was carried out using a sustainable method in the presence of multiple antibiotics in growing media (**Figure 1**). The majority of the isolates were identified as *Pseudomonas* sp. based on their biochemical properties. The most resistant strain, GZB16/CEES1, exhibited fluorescent green pigmentation on the King’s B media plate under ultraviolet light (see **Figure 1B**). Among the 20 bacterial isolates, GZB16 showed resistance to all 17 (100%) different antibiotics, while the remaining isolates displayed varying levels of resistance to the administered antibiotics, with the lowest being 35%. A study by Luczkiewicz et al. reported that *Pseudomonas* sp. possesses or can acquire a wide range of antibiotic resistance mechanisms (Luczkiewicz et al., 2015). They emphasized the need for careful monitoring of *Pseudomonas* for the potential dissemination of drug-resistance genes.

In some other previous research has reported antibiotic resistance among *Pseudomonas* sp. isolates from hospital wastewater treatment plant samples (Santoro et al., 2015). Another study identified antibiotic-resistant *P. aeruginosa* isolated from wastewater channels containing wastewater from different hospitals (Slekovec et al., 2012). Zhou et al. also reported isolating *Pseudomonas* sp. (5%) from activated sludge with an antibiotic-resistance gene (Zhou et al., 2019). It is worth noting that wastewater treatment plants have been found to serve as reservoirs for antibiotic resistance-carrying genes among bacteria (Zhang et al., 2019).

**Figure 1C** shows that the isolated bacterial strains produced the pyocyanin pigment on both the media plate and liquid culture. The phenazine biosynthesis pathway in *Pseudomonas* makes pyocyanin. This protein is involved in infection and virulence, which makes it an interesting target for finding new drugs that fight infections (Xie and Xie, 2019). The bacterial strain GZB16 was characterized using both biochemical and molecular methods. The test showed that it worked for citrate utilization, lipase, urease, catalases, and oxidases but not for methyl red, Voges-Proskauer, lysine decarboxylase, or phenylalanine deaminase. The genetic characterization of strain GZB16 was performed using 16S rDNA sequencing (**Figure 4A**). BLASTn analysis revealed that the observed 99% similarity of the JQ267512-1 sequence belonged to the *Pseudomonas aeruginosa* strain. The phylogenetic tree was developed based on the maximum likelihood of similar sequences (**Figure 4B**). Pathogenic and similar phylogenetic features of *P. aeruginosa* were found and studied at the cleanup site (Kaszab et al., 2016). Additionally, a study identified a multidrug-resistant bacterial phase carrying the *P. aeruginosa* strain (Jamal et al., 2017).

Previously, Young et al. (2019) revealed that the production of β-lactamase leads to imipenem resistance in *P. aeruginosa*. A study showed that a *P. aeruginosa* strain isolated from dog swabs resisted 6–10 antimicrobial agents through integrons (Lin et al., 2012). Zhao et al. discovered P. aeruginosa in mink and found resistance to ten different antibiotics (Zhao et al., 2018). In a different study, *Pseudomonas* strains were completely sensitive to gentamicin, clindamycin, and ofloxacin, but they were 90–100% resistant to penicillin, rifampin, and sulphamethoxazole (Odjadjare et al., 2012). A report by another researcher demonstrated that antibiotic-resistant bacteria can survive in hospital-generated wastewater (Kalaiselvi et al., 2016). An antibiotic-resistant P. aeruginosa strain was identified in an Iranian burn medical facility (Ebrahimpour et al., 2018). Yewale et al. (2020) reported wastewater as a reservoir of antibiotic-resistance genes and multidrug-resistant bacteria. They analyzed the antibiotic resistance in microbial diversity from the river wastewater of Pune, India. Strain GZB 16 may be naturally resistant to multiple drugs or have gained this through plasmid transfer and conjugation with other drug-resistant bacterial strains. The drug-resistant plasmid DNA was obtained from the Pseudomonas aeruginosa strain GZB16/CEES1 (**Figure 6**).

Researchers have found a pattern similar to this. The trans-conjugation method (Sikandar and Shahida, 1994) can be used to move the metal resistance gene of P. aeruginosa to an E. coli strain (recipient) after eight hours of incubation. Genetic recombination, including horizontal gene transfer between different bacterial populations, is essential for adaptation in adverse environmental conditions. In soil and water ecosystems, transferring genetic elements through plasmids is critical to advancing bacterial populations (Kozdroj, 1994). Conjugative gene transfer via horizontal plasmids is the primary way antibiotic-resistance genes are spread among bacterial populations. Controlling multidrug-resistant bacterial pathogens severely threatening immunosuppressed hospital patients is particularly challenging. Few studies have investigated the frequency of plasmid transfer in soil and open field conditions and the recovered conjugated plasmids from bacteria isolated from polluted soil samples (Lilley et al., 1996; Van Elsas and Bailey, 2002). Determining how bacteria adapt to different environments through horizontal gene transfer via plasmids in naturally colonizing bacteria in semi-natural conditions is crucial for understanding how bacteria do this. One of the key factors to consider during such studies is the potential risks associated with the proliferation of resistance genes among bacteria, which may threaten both animal and human health (Van Elsas and Bailey, 2002). Additionally, it is essential to identify the environmental samples where these bacteria reside, as they often serve as reservoirs for antibiotic-resistance genes (Saile and Koehler, 2006).

This study used a temperature-variation incubation technique for the plasmid curing experiment. We used 5% acridine orange in the process, which is more concentrated than the 3% used in previous research to cure the plasmid of the *Pseudomonas* strain (Bhattacharya et al., 2000). This gave us more accurate results. Another study reported 100% plasmid curing of *E. coli* isolates from drinking water using acridine orange (Diab et al., 2002). The antibiotic susceptibility pattern (**Figure 6**) confirmed our observation: the antibiotic resistance gene had completely disappeared in our study. This was done by changing the temperature of the cells from 35 to 45°C and adding 5% acridine orange.

This study aimed to discover how exopolysaccharides (EPS) help the bacteria grown in 5% sucrose form biofilms. As a readily available carbon source and an inherent feature of the isolates’ native habitat, sucrose fulfilled two functions in this scenario. One well-established element in forming biofilms is the generation of extracellular matrix (EPS), which plays a major role in both the initial adhesion stage and the subsequent maturation of biofilms (Flemming and Wingender, 2010). Bacterial cells are known to be encapsulated by EPS matrices, which give the biofilm structural integrity and act as a barrier against adverse conditions such as desiccation, antimicrobials, and the host immune system (Okshevsky and Meyer, 2015). The study found that strain GZB 6 was the most active producer of extracellular solids (EPS), with a 21 µg/ml concentration. This large EPS output indicates that, given the protective advantages provided by the EPS, GZB 6 might have increased virulence and survivability. The pathogenic potential of strains GZB 8, 15, and 16 and their consequent high EPS production (20 µg/ml) further highlights the significance of EPS in biofilm resilience. It is important to remember that the EPS’s structural makeup and physical characteristics also play important roles in determining the biofilm’s strength and resilience; EPS amount alone does not determine these characteristics (Mishra et al., 2023). It is crucial to conduct further research into these strains’ EPS composition and biofilm architecture to understand the implications of high EPS generation. The EPS production profiles reported provide valuable information for developing anti-biofilm strategies. By focusing on the prominent producers, such as GZB 16/CEES1, the formation and persistence of harmful biofilms may be diminished. Future studies should concentrate on the structural characteristics of the biofilms formed, the molecular mechanisms of EPS synthesis in these strains, and the creation of treatments to regulate biofilm formation in clinical settings.

### 4.1 Bacterial growth and pyocyanin synthesis

The data reveals a robust connection between the bacterial biomass amount and the broth culture’s growth progression. Moreover, a correlation exists between the quantity of bacterial biomass and pyocyanin generation, a virulence factor produced by Pseudomonas species. The substantial growth observed under optimal nutritional conditions aligns with the expected logarithmic phase of bacterial expansion. However, the subsequent plateau or lack of progress suggests the transition of the bacterial culture into the stationary phase, in which growth ceases due to the depletion of nutrients, accumulation of waste products, or other limiting factors (Bremer and Dennis, 2008). The relationship between pyocyanin production and bacterial proliferation is particularly noteworthy. Pyocyanin concentration was highest after 72 and 96 hours of incubation during the transition from exponential to stationary growth phase. These results suggest that pyocyanin production is associated with a specific phase in the bacterial life cycle, possibly as a response to changing conditions in the culture medium. The data imply that the peak production of pyocyanin occurs when the bacterial culture ceases to grow, potentially as a strategy to gain a competitive advantage or ensure survival. Notably, the maximum pyocyanin concentration does not align with the highest biomass level. The difference can be ascribed to the metabolic changes bacteria experience when adjusting to stationary phase settings. In this stage, the bacterium may increase the production of secondary metabolites, such as pyocyanin, either as a response to stress or as a tactic to prepare the environment for survival (Bastos et al., 2022). The results of this study have implications for understanding the mechanisms of *Pseudomonas* growth and pyocyanin synthesis, which is crucial in clinical settings where Pseudomonas infections are known for their persistent nature and resistance to treatment. The timing of pyocyanin synthesis may offer valuable information for determining the optimal timing of interventions to specifically target the period preceding the peak production of virulence factors. These results contribute to our understanding of bacterial growth rates and the generation of secondary compounds, which may hold practical relevance in medical therapy and industrial microbiology.

### 4.2 Anti-candida activities

The study reveals a noteworthy discovery on the antifungal properties of *Pseudomonas aeruginosa*, achieved through the synthesis of pyocyanin, a compound that demonstrates anti-*Candida* effects. The interesting finding is that pyocyanin exhibits dose-dependent inhibition of *Candida albicans* growth, resulting in a peak inhibition zone of 24 mm when the concentration is 3.2 µg/ml. The expanding area of bacterial growth inhibition observed with higher concentrations of pyocyanin provides evidence for its potential as a medicinal agent. The reduced ability of *Candida albicans* to survive after 96 hours of incubation is associated with the peak levels of pyocyanin synthesis, suggesting that this is the crucial period when *Pseudomonas aeruginosa* exerts its strongest antifungal effect. Nevertheless, as *Pseudomonas* reaches the declining phase, the generation of pyocyanin diminishes, leading to a concomitant rise in *Candida* survival. These findings indicate that the antifungal properties of pyocyanin are strongly associated with the active proliferation stage of the bacteria.

Furthermore, the study illustrates the influence of pyocyanin on the creation of biofilms, which is an essential element in the pathogenicity of *Candida albicans*. The total suppression of biofilm development through pyocyanin and the 50% decrease in *Candida* growth when exposed to this pigment highlights the promising prospect of addressing infections linked with biofilms (**Supplementary Figures 3A, B**). The antagonistic relationship between *Pseudomonas* and *Candida* is visually confirmed through direct interaction, as observed by scanning electron microscopy, where bacterial cells counteract the fungal cells (**Figure 9**), control image of candida biofilm can compared from the previous reported research (Oves et al., 2017, Oves et al., 2018). This study has two implications. Firstly, it indicates that pyocyanin exerts a significant inhibitory impact on *Candida albicans*, which could be utilized to create antifungal medications. Furthermore, *Pseudomonas aeruginosa* capacity to hinder biofilms’ production and dismantle already established biofilms could be an innovative strategy for addressing fungal infections, especially those unresponsive to conventional antifungal therapies. Additional investigation into the molecular mechanisms that underlie pyocyanin’s antifungal activity could lead to the development of novel treatment strategies for *Candida* infections.

## 5 Conclusion

The extensive investigation conducted on the bacterial isolates obtained from the Hindan River has brought to light the serious issue of antibiotic resistance in environmental samples, particularly in the Pseudomonas species. The remarkable strain GZB16 highlights the challenges in treating infections caused by these bacteria, as it is not only virulence factor-producing and resistant to several medications but also possesses other complex characteristics. The results corroborate the role of wastewater as a reservoir for genes and bacteria resistant to antibiotics and are consistent with other studies in this field. This underscores the importance of closely monitoring and managing the spread of these infections, as doing so could significantly impact public health. The study’s findings regarding the bacteria’s ability to form biofilms, especially the EPS synthesis by strain, highlight the intricate nature of bacterial survival strategies. The timing of therapeutic interventions in clinical settings is influenced by the strong correlation observed between bacterial biomass, growth progression, and pyocyanin synthesis. To break the cycle of infection and resistance, targeting the bacterial response to environmental stress may be necessary, as suggested by the peak in pyocyanin production during the stationary phase. Further pyocyanin is used to control the growth of *Candida albicans*. In summary, this study raises awareness of the potential dangers posed by these bacteria while also contributing to our understanding of antibiotic resistance and virulence characteristics in environmental isolates of Pseudomonas. Most of the virulence gene was encoded by the plasmid, it was confirmed by plasmid curing assay. It highlights the need for innovative approaches to effectively treat infections caused by microorganisms resistant to multiple drugs and slow the spread of resistance genes. The study serves as a call to action for the clinical, scientific, and environmental communities to confront the growing problem of antibiotic resistance in a comprehensive and coordinated manner.

## 6 Summary

The current study sheds significant light on the intricate mechanisms that govern antibiotic resistance and virulence factor production in *Pseudomonas aeruginosa*, a notably problematic bacterial pathogen. This research underscores the profound environmental impact of multidrug-resistant bacteria, emphasizing the urgency for innovative approaches to tackle such formidable pathogens. A key aspect of this study is its focus on the environmental implications of antibiotic resistance. The presence of *Pseudomonas aeruginosa* in soil samples from an industrial area highlights the broader issue of environmental contamination with resistant bacteria. This contamination poses a public health risk and stresses the ecosystem.

Moreover, the findings of this study are critical in understanding the development of resistance mechanisms and virulence factors in Pseudomonas aeruginosa. Such understanding is fundamental for designing new antimicrobial therapies, especially in an era where traditional antibiotics are increasingly becoming ineffective against resistant strains. The study also offers valuable insights into the battle against *Candida albicans*, a common fungal pathogen. The potential of developing novel antimicrobial treatments, as suggested by the study, could significantly impact the management of Candida infections, which are particularly problematic in immunocompromised patients. In summary, the research presented in this study contributes to the scientific understanding of antibiotic resistance and virulence in *Pseudomonas aeruginosa*. It provides a crucial foundation for future innovations in antimicrobial therapy. It highlights the necessity of continued research and development in this field to address the escalating threat posed by multidrug-resistant organisms in healthcare and environmental settings.

## Data availability statement

The datasets presented in this study can be found in online repositories. The names of the repository/repositories and accession number(s) can be found below: EBI.ac.uk, LN736035.

## Author contributions

MO: Writing – review & editing, Writing – original draft, Software, Resources, Methodology, Investigation, Formal analysis, Data curation, Conceptualization. MShK: Writing – original draft, Writing – review & editing, Visualization, Resources, Funding acquisition, Data curation. MA: Writing – review & editing, Writing – original draft, Visualization, Validation, Supervision, Project administration, Funding acquisition. MSaK: Writing – review & editing, Writing – original draft, Visualization, Supervision, Resources, Formal analysis.
