# Supplementary material for: Antifungal potential of multi-drug-resistant Pseudomonas aeruginosa: harnessing pyocyanin for candida growth inhibition
Source: Front Cell Infect Microbiol. 2024 May 22;14:1375872. doi: 10.3389/fcimb.2024.1375872 (PMC11155300; doi:10.3389/fcimb.2024.1375872)
Supplement: Supplementary file 1 [file DataSheet_1.docx]

# Supplementary Material

Antifungal Potential of Multi-Drug-Resistant Pseudomonas aeruginosa: Harnessing Pyocyanin for Candida Growth Inhibition

Mohammad Oves^1,*^,Mohd Shahnawaz khan^2^, Majed Al-Shaeri^3,^ Mohammad Saghir Khan^4^,

^1^Centre of Excellence in Environmental Studies, King Abdulaziz University, Jeddah, 21589, Kingdom of Saudi Arabia

^2^ Department of Biochemistry, College of Science, King Saud University, Riyadh, Saudi Arabia

^3^Department of Biological Science, Faculty of Science, King Abdulaziz University, Jeddah, 21589, Kingdom of Saudi Arabia.

^4^Department of Microbiology, Faculty of Agricultural Science, Aligarh Muslim University, Aligarh, India

**2.3.2. Biochemical characteristics**

We used the microbiological and biochemical methods described in Bergey's Manual of Determinative Bacteriology [24] to characterize the bacterial cultures. These included the indole reaction, citrate utilization, catalase production, H2O2 production, nitrate reduction, sugar fermentation, and starch and gelatin hydrolysis tests. Each bacterial isolate was cultured in a sterile nutrient broth for 48 hours for the indole test at 35 °C. The crimson ring was a positive indole reaction after incubating the acquired culture broth with 2-3 drops of Kovac's reagent. Each isolate was inoculated into MR-VP broth and incubated for 48 hours at 35 °C. As an indication, a solution of methyl red was amended, and the formation of red color was considered a methyl red-positive bacterial strain. In addition, test strains were introduced into sterile MR-VP broth for 48 hours at 35 °C to distinguish the MR-VP outcomes. The freshly obtained culture was combined with Barrit's reagent, and the red color indicated a positive Voges-Proskauer (VP) test.

The Simmon's citrate agar plates with spot inoculation with the test strain were incubated at 35 °C for 48 hours, and the color change from green to blue indicated positive test results. The color change means the citrate utilization by the bacteria from green to blue. The test isolates were inoculated in broth for the catalase reaction and incubated at 30 °C for 48 hours. After incubation, 3 percent H2O2 was added to the slide with culture, and bubble formation was seen within minutes due to free radical formation. For the nitrate reduction assay, test isolates were put into autoclaved trypticase nitrate broth tubes and left to sit at 35 °C for 48 hours. After testing how well the bacterial cultures could use carbohydrates, each strain was added to a sterile fermentation broth with three sugars added at a concentration of 5 g/l each: glucose, sucrose, and mannitol. The mixture was then left to grow at 30 °C for 48 hours. The extent of acid or gas production was then observed. To test how well starch was broken down, 10 µl of each bacterial strain was spread out on autoclaved starch agar plates. The plates were then left at 30 °C for 48 hours and put in an incubator. After that, an iodine solution was put on the plates to break down the starch. The starch had been broken down because of a clear hydrolysis zone around the bacteria's growth. In addition, test tubes containing sterile broth and 12% gelatin were inoculated with the test isolate and incubated at 4 °C for 30 minutes. The liquidity of the gelatin tubes was then analyzed due to the positive test for the gelatin hydrolysis reaction that liquefied the gelatin tubes.


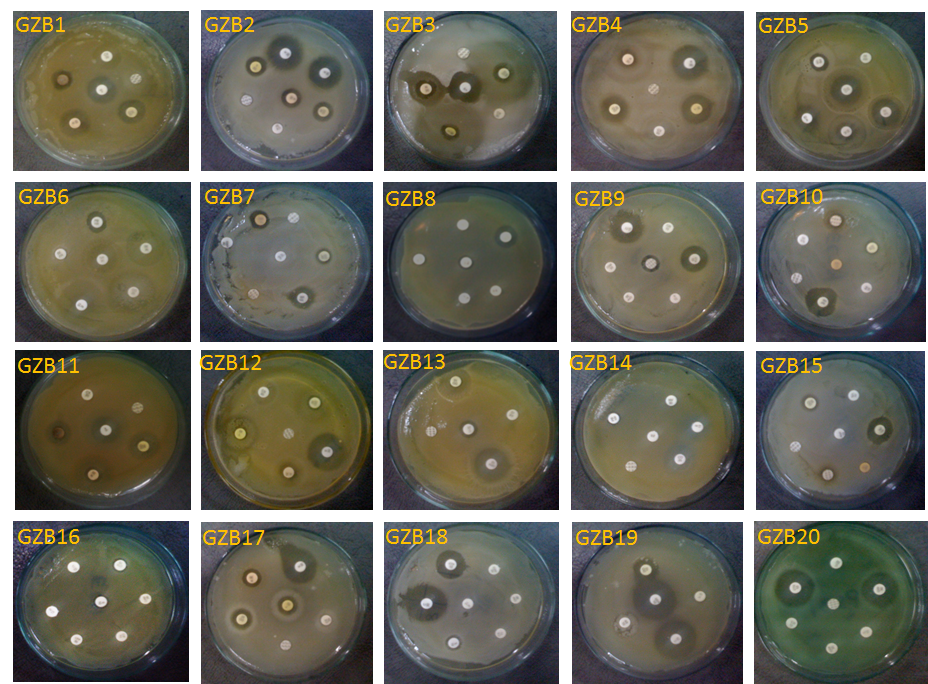


Figure .1. Bacterial isolates GZB1 to GZB20 and show antibacterial disc diffusion pattern (SI)

>ENA|LN736035|LN736035.1 Pseudomonas aeruginosa partial 16S rRNA gene, strain CEES1 AGCCAGGTGGTTGAGCCCAGGTCACAATTAATAGTTTGGGCCTCAACCGGACCCCTCCTCCAACAAGGGAAACGGGGGGGAGGGGGGGGGAATTATCTTTTGGGGAGACGCGCGAAATGTTTATTATTTGGGTTTAGTTCCAGATTGAGATCGAACGCCGGGGGTCAGGACAAACACTTTGCAACTCAAGCCGAAGGAGGTCCCCCTTTCCTCTCGCTCCAGCGACGGACGGGTGAGTAATGTCTAGCAATCTACCTGATACTGGGGGGATAACACCCGAAAACGGGCGCTCAATACGGCATACCTCCGCAAGGCCAAAGCGGGGAACATTCGGACCTCACGCCATCAGATGGGCCCAGATGGGATTAGCTAGTAGGTGGGGTAAAGGCTCACCCAGGCGACGATCCGTAGCTGGTCTGAGAGGATGATCAGTCACACTGGAAGTGAGACACGGTCCAGACTCCTACGGGAGGCAGCAGTGGGGAATATTGCACAATGGGCGAAAGCCTGATCCAGCCATGCCGCGTGCGTGAAGAACGTCTTCGGATTGTAAAGCACCTTAAGTGAGGAGGAAGGGCAGTAAGTTAATACCTTGATCTTTTGACGTTACCAGCAGAAGAAGCACCGGCTAACTCCGTGCCAGCAGCCGCGGTAATACGGAGGGTGCAAGCGTTAATCGGAATTACTGGGCGTAAAGCGCGCGCAGGTGGTTCAGCAAGTCGGTATGTGACTTCCCCGGGCTCAACCTGGGAACTGCATCCGAAACTAGCGAGCTAGAGTCTGGTAGAGGGGGCTAGAACTTCCGCTGTCGCGGTGTATGCGTAGGATAGTTTTTTCCCAGGACCCTGGCCTCCCCCTCGCGCAGCCTGC

Figure 2 Bacterial strain GZB 16 or CEES1 of *P. aeruginosa* FASTA sequence obtained from the 16S rRNA gene sequencing analysis. (SI2)


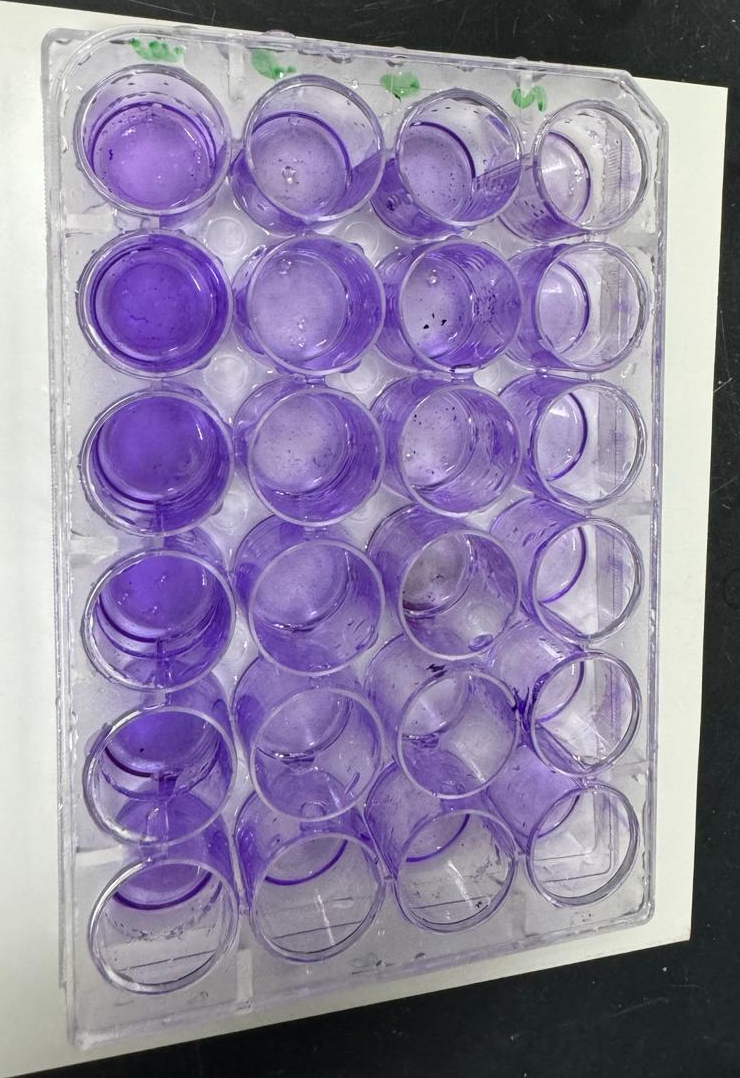


Figure 3a: Candida albicans biofilm testing in culture plate separately without treatment.


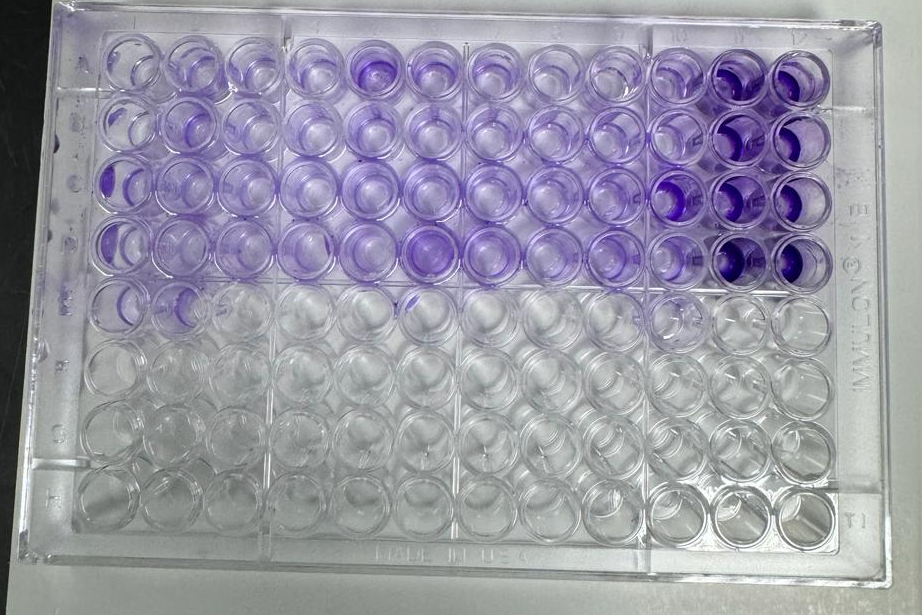


Figure 3 b: Antibiofilm study of *candida albicans* in the presence of different concentrations of pyocyanin in the 96 well plates clearly shows by crystal violet staining

Table 1. Antibiotics and their potency are used in the present study.

| Antibiotics | Disc code | Potency (µgdisc^-1^) |
| --- | --- | --- |
| Amoxicillin | Am | 30 |
| Chloramphenicol | C | 25 |
| Ciprofloxacin | Cf | 30 |
| Cloxacillin | Cx | 30 |
| Doxycycline | Do | 30 |
| Erythromycin | E | 10 |
| Gentamycin | G | 30 |
| Kanamycin | K | 30 |
| Methicillin | M | 30 |
| Nalidixic acid | NA | 30 |
| Nitrofurantoin | Nf | 30 |
| Norfloxacin | Nx | 10 |
| Novobiocin | Nv | 30 |
| Penicillin G | P | 10 |
| Carbapenem  Imipenem | Cp  Im | 30  30 |
| Rifampicin | R | 30 |
| Tetracycline | T | 30 |

Source: Hi-media Pvt. Ltd. Mumbai, India

Table 2. Morphological and biochemical characteristics of GZB16/CEES1 strain of *Pseudomonas aeruginosa*

| Characteristics | Features | Results |
| --- | --- | --- |
| *Morphology* | Gram reaction | -ve |
|  | Shape | Rod |
|  | Colony | Oval |
| *Pigmentation* | Blue green | Pyocyanin |
| *Metabolism* | Type | Facultative anaerobe |
| *Swarming* | Flagella motility | +ve |
| *Enzyme assay* | Lipase | +ve |
|  | Catalase | +ve |
|  | Oxidase | +ve |
|  | Phenylalanine deaminase | -ve |
|  | Lysine decarboxylase | -ve |
| *Hydrolysis* | Gilatin | +ve |
|  | Urea | +ve |
| *Fermentation* |  |  |
| *Mannitol* | Acid production | +ve |
| *Lactose* | Acid production | -ve |
| *Glucose* | Acid production | -ve |
| *Sucrose* | Acid production | -ve |
| *Glucose* | Acid production | -ve |
| *IMVic test* | Indole | -ve |
|  | Methyl red | -ve |
|  | Voges-Proskaeur | -ve |
|  | Citrate | +ve |

Table 3. Antibiotic resistance/sensitivity profile of bacterial diversity recovered from the Ghaziabad city outskirts of Hindon river muddy soil samples.

| Bacterial strains | Zone of Inhibition (mm) | | | | | | | | | | | | | | | | | Resistant pattern | R (%) | S (%) |
| --- | --- | --- | --- | --- | --- | --- | --- | --- | --- | --- | --- | --- | --- | --- | --- | --- | --- | --- | --- | --- |
|  | Am  (30) | Cf  (30) | Cx  (30) | C  (25) | E  (10) | G  (30) | K  (30) | M  (30) | NA  (30) | P  (10) | Pb  (50) | R  (30) | Nf  (30) | Nx  (10) | Do  (30) | T  (30) | Nv  (30) |  |  |  |
| GZB1 | R | 34 | R | 15 | 18 | 19 | 20 | R | 16 | R | R | 15 | R | 34 | 20 | R | R | Am,Cx,M,P,Pb,Nf,T, Nv | 47 | 53 |
| GZB 2 | R | 40 | R | 20 | 18 | 18 | 20 | R | 16 | R | 14 | 13 | R | 30 | 19 | R | R | Am,Cx,M,P,Nf,T,Nv | 41 | 59 |
| GZB 3 | 18 | 40 | R | 26 | 30 | 28 | 21 | R | 13 | R | R | R | R | 36 | 32 | 22 | 15 | Cx, M, P, Pb, R, Nf | 35 | 65 |
| GZB 4 | 18 | 40 | R | 26 | 30 | 28 | 21 | R | 13 | R | R | R | R | 36 | 32 | 22 | 15 | Cx, M, P, Pb, R, Nf | 35 | 65 |
| GZB 5 | R | 30 | R | 15 | 20 | 20 | 20 | R | 15 | R | R | R | R | 18 | 26 | R | R | Am,Cx,M,P,Pb,Nv,Nf,R,T | 52 | 48 |
| GZB 6 | R | 42 | R | 26 | 35 | 26 | 22 | R | R | R | R | R | R | 23 | 18 | 16 | 17 | Am, Cx, M, NA, P, Pb, R, Nf | 47 | 53 |
| GZB 7 | R | 36 | R | 13 | 14 | 21 | 19 | R | R | R | R | R | R | 27 | R | R | R | Am,Cx,M,NA,P,Pb,R,Nf,Do,T,Nv | 64 | 36 |
| GZB 8 | R | 28 | R | 15 | 12 | 22 | R | R | R | R | R | R | R | 18 | 15 | R | R | Am,Cx,E,K,M,NA,P, Pb,Nf,R,T,Nv | 70 | 30 |
| GZB 9 | R | 36 | R | 21 | 17 | 29 | R | R | 15 | R | R | R | R | 31 | R | R | R | Am,Cx,K,M,,P,Pb,R,Nf,Do,T,Nv | 64 | 36 |
| GZB 10 | R | 37 | R | 15 | R | 26 | R | R | R | R | R | R | R | 33 | R | R | R | Am,Cx,E,K,M,NA,P,Pb,R,Nf,Do,T,Nv | 76 | 24 |
| GZB 11 | R | 39 | R | 16 | R | 25 | R | R | R | R | R | R | R | 36 | R | R | R | Am,Cx,E,K,M,NA,P,Pb,R,Nf,Do,T,Nv | 76 | 24 |
| GZB 12 | R | 44 | R | 20 | 15 | 20 | R | R | R | R | R | R | R | 35 | R | R | R | Am,Cx,K,M,NA,P,Pb,R,Nf,Do,T,Nv | 70 | 30 |
| GZB 13 | R | 36 | R | 21 | 17 | 29 | R | R | 15 | R | R | R | R | 31 | R | R | R | Am,Cx,K,M,,P,Pb,R,Nf,Do,T,Nv | 64 | 36 |
| GZB 14 | R | 28 | R | R | R | 25 | 24 | R | R | R | R | 13 | R | 25 | R | R | R | Am,Cx,C,E,M,NA,P,Pb,Nf,Do,T,Nv | 70 | 30 |
| GZB 15 | 18 | 25 | R | R | 12 | 21 | 13 | R | R | R | R | R | R | 15 | 13 | R | R | Am,Cx,C,E,M,NA,P,Pb,R,Nf T,Nv | 70 | 30 |
| GZB 16 | R | R | R | R | R | R | R | R | R | R | R | R | R | R | R | R | R | Am,Cf,Cx,C,E,G,K,M,NA,P,Pb,R,Nf, Nx, Do,T, Nv | 100 | 0 |
| GZB 17 | R | 42 | R | 26 | 35 | 26 | 22 | R | R | R | R | R | R | 23 | 18 | 16 | 17 | Am, Cx, M, NA, P, R, Pb, Nf | 47 | 53 |
| GZB 18 | R | 30 | R | R | R | 20 | 20 | R | R | R | R | R | R | 20 | R | R | R | Am,Cx,C,E,M,NA,P,Pb,R,Nf, Do,T,Nv | 76 | 24 |
| GZB 19 | R | 28 | R | 15 | R | 22 | R | R | R | R | R | R | R | 18 | 15 | R | R | Am,Cx,E,K,P,M,NA,Pb,Nv,Nf,R,T | 70 | 30 |
| GZB 20 | R | 30 | R | 15 | 20 | 20 | 20 | R | 15 | R | R | R | R | 18 | 26 | R | R | Am,Cx,M,P,Pb, Nf,R,T, Nv | 52 | 48 |
